# Pretreatment quality of life and survival in patients with lung cancer: a systematic review and meta-analysis

**DOI:** 10.1186/s12885-024-12267-w

**Published:** 2024-04-18

**Authors:** Taro Okayama, Katsuyoshi Suzuki, Shinichiro Morishita, Junichiro Inoue, Takashi Tanaka, Jiro Nakano, Takuya Fukushima

**Affiliations:** 1 Division of Rehabilitation Medicine, Shizuoka Cancer Center, Shizuoka, Japan; 2 Department of Physical Therapy, School of Health Science, Fukushima Medical University, Fukushima, Japan; 3 Division of Rehabilitation Medicine, Kobe University Hospital International Clinical Cancer Research Center, Kobe, Japan; 4 Department of Rehabilitation, Hyogo Medical University Hospital, Nishinomiya, Japan; 5 Faculty of Rehabilitation, Kansai Medical University, Osaka, Japan

**Keywords:** Quality of life, Lung cancer, Mortality risk, Systematic review, Meta-analysis

## Abstract

**Background:**

Although many studies have explored the correlation between quality of life and survival, none have reported this relationship for specific cancers assessed at distinct time points. This meta-analysis aimed to investigate the impact of pretreatment Global Quality of Life (QOL) and functioning QOL, including physical, social, role, emotional, and cognitive QOLs, on mortality risk in patients with lung cancer.

**Methods:**

A literature search was conducted across the Cumulative Index to Nursing and Allied Health Literature (CINAHL), Scopus, and PubMed databases for articles published between their inception and December 2022. Subsequently, 11 studies were selected based on predefined eligibility criteria to investigate the relationship between pretreatment QOLs and mortality risk in patients with lung cancer.

**Results:**

Pretreatment global, physical, social, role, and emotional QOLs were significantly associated with mortality risk as follows: Global QOL (hazard ratio [HR] = 1.08 95% confidence interval [CI] = 1.03–1.13); Physical QOL (HR = 1.04 95% CI = 1.02–1.05); Social QOL (HR = 1.02 95% CI = 1.01–1.03; Role QOL (HR = 1.01 95% CI = 1.01–1.02); Emotional QOL (HR = 1.01 95% CI = 1.00–1.03).

**Conclusions:**

These findings underscore the importance of early QOL assessment after diagnosis as well as early provision of physical, social, and psychological support accommodating each patient’s demands.

**Trial registration:**

The International Prospective Register of Systematic Reviews registration number CRD42023398206, Registered on February 20, 2023.

**Supplementary Information:**

The online version contains supplementary material available at 10.1186/s12885-024-12267-w.

## Background

The number of patients with lung cancer has been increasing in recent years owing to global aging of the population and advances in cancer treatments. Approximately 2.2 million cases and 1.8 million deaths (18% of all sites) [1] occur annually worldwide, which is significantly higher than the 930,000 annual deaths (9.4% of all sites) of secondary colorectal cancer. Advances in screening techniques and agents have extended the survival of patients with lung cancer, especially non-small cell lung cancer, although the 5-year survival rate for patients diagnosed in 2010–2014 remains 20–30% [2], the third lowest survival rate after that for pancreatic and liver cancer. More than half of the patients with lung cancer have distant metastases at diagnosis [3], and even when surgery is performed as the initial treatment, the recurrence rate is high [4].

In response to this situation, many patients receive palliative treatment, which means that QOL assessment is especially important for patients with lung cancer. The World Health Organization (WHO) defines QOL as " individuals' perception of their position in life in context of the culture and value systems in which they live and in relation to their goals, expectations, standards, and concerns. " [5]. QOL assessment consists of Global QOL and functioning QOL, which pertains to specific functions such as Physical, Role, Cognitive, and Emotional. QOL assessment helps to understand the impact of disease and treatment on patients' overall lives [6–8], evaluate the effects of anticancer treatment and supportive care [9, 10], detect side effects and complications [11], notice the differences between symptoms from the patient’s and healthcare provider’s points of view [12], and guide long-term follow-up [13, 14]. In addition, QOL assessment is a useful tool for predicting prognosis.

Several systematic reviews, pooled analyses, and meta-analyses on QOL and survival have been published [15–20]. These studies have mostly shown positive results in predicting prognosis. However, various problems have been pointed out in these studies, such as no distinction between cancer types [15], no distinction of cases before, during, and after treatment [19], and the use of only the European Organization for Research and Treatment of Cancer Quality of Life Questionnaire-Core 30 (EORTC QLQ-C30) in QOL assessment [17, 20]. In addition, the relationship between other functional domains of QOL and prognosis is not well understood because previous studies have focused on global and physical QOL, which are more related to prognosis. Qi et al. [21] in a study of 420 patients with advanced lung cancer reported that pretreatment QOL and body mass index were significant prognostic factors. Sloan et al. [22] reported that QOL at diagnosis can be an independent prognostic factor. However, Qi et al. and Sloan et al. assessed QOL using single items UNISCALE and one of the Lung Cancer Symptom Scales, respectively, which raises questions about detailed assessment. This means that the relationship between pretreatment QOL and the mortality risk in patients with lung cancer has not been adequately studied and a certain view has not been reached. This suggests that the relationship between pretreatment QOL and the mortality risk in patients with lung cancer has not been adequately studied.

Given the limitations of the previous studies, there is an urgent need to evaluate the relationship between various domains of QOL and survival in patients with lung cancer to enable application of appropriate interventions to improve prognosis. Thus, this systematic review and meta-analysis aims to clarify the significance of each QOL domain for mortality risk and provide information to inform future clinical practice and interventions.

## Methods

This systematic review and meta-analysis was registered in the International Prospective Register of Systematic Reviews (registration number CRD42023398206) [23] and followed the Preferred Reporting Items for Systematic Reviews and Meta-Analysis guidelines [24].

### Data searches and sources

A systematic search was conducted using the PubMed/MEDLINE, CINAHL, and Scopus databases from inception to December 2022. The search strategies used in each database included QOL, EORTC QLQ-C30 [25], Medical Outcomes Study 36-Item Short-Form Health Survey (SF-36) [26], Functional Assessment of Cancer Therapy-General (FACT-G) [27], cancer, neoplasm, tumor, mortality, survival, relapse, and recurrence. The details of the search strategy used for each database are provided in the Supplementary Appendix.

### Study eligibility criteria and study selection

The eligibility criteria were as follows: 1) original human studies, 2) observational studies, 3) studies published in English, 4) studies on patients with lung and malignant pleural mesothelioma, and 5) studies that examined the association between pretreatment QOL and mortality. The exclusion criteria were as follows: 1) studies involving patients other than those with lung or malignant pleural mesothelioma, 2) studies examining the association between QOL during or after treatment and mortality, and 3) studies examining the association between symptoms and mortality. After removing duplicates, seven reviewers independently assessed the study eligibility by reviewing the titles and abstracts of all potential citations according to the eligibility criteria. Full-text articles were retrieved for review if there was evidence that they met the eligibility criteria, or if there was insufficient information in the abstract or title to make a decision. The final inclusion of eligible observational studies was determined at consensus meetings attended by all the authors.

### Data extraction

Two reviewers (TO and TF) extracted the data. The following data were extracted from each included study: 1) last name of the first author, 2) year of publication, 3) nationality, 4) number of patients, 5) sex, 6) age, 7) histology, 8) clinical stage, 9) cancer treatment, 10) QOL, 11) QOL domains, 12) follow-up period, 13) covariates adjusted in the multivariate analysis, 14) number of deaths, and 15) risk estimates for mortality (hazard ratio [HR] and 95% CI). When several different models of multivariate analysis were available, we used the results from multivariate models with the most complete adjustments for potential confounders.

### Quality assessment

The quality of studies, including their risk of bias, was assessed using the Newcastle–Ottawa scale [28]. This tool includes the following eight domains: representativeness of the exposed cohort; selection of the non-exposed cohort; ascertainment of exposure; demonstration that the outcome of interest was not present at baseline; comparability of cohorts based on design or analysis; assessment of outcomes; whether follow-up was long enough for outcomes to occur; and adequacy of the cohort follow-up. Two trained reviewers (TO and TF) scored each item based on these criteria [28]. Potential disagreements were resolved through consensus meetings involving all authors.

### Data analysis

Risk estimates of total mortality were analyzed in relation to pretreatment global, physical, emotional, role, cognitive, and social QOL. We used adjusted HRs and 95% CI in the multivariate analysis as measures of the effect size for all studies. Univariate HRs were used only when reported, but not multivariate HR. For inverse variance-weighted means, the natural log of HR was used, and the standard error was calculated using a random-effects model. Heterogeneity was assessed using the *I*^*2*^ statistic. All statistical analyses were performed using the Review Manager version 5.1 (RevMan; The Cochrane Collaboration, London, UK).

## Results

**Fig. 1:**
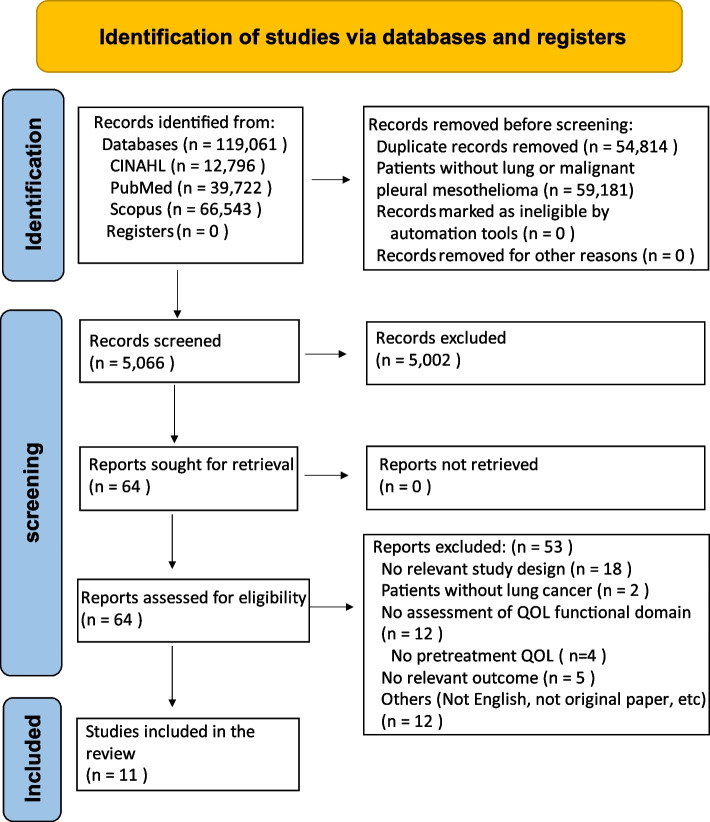
Study flow diagram of the selection process

The database search yielded 119,061 articles, which were reduced to 5066 articles after excluding duplicates. These 5066 articles were screened for titles and abstracts, after the exclusion of 5002 studies due to irrelevant study designs or discrepancies regarding the population or outcomes. A full-text review was conducted on the remaining 64 articles, and 53 studies were excluded due to irrelevant study design or outcomes, non-lung cancer, different languages, non-original articles, and finally 11 articles were determined to be suitable for meta-analysis (Fig. 1).

### Study characteristics

The characteristics of the 11 studies that met the inclusion criteria are summarized in Table 1. These studies was published between 2000 and 2022. Their sample sizes ranged from the smallest (50 patients) in the study by Erdem et al. [29] to the largest 2892 patients in the study by Badaoui et al. [30]. These patients had non-small cell lung cancer [29–33], small cell lung cancer [34], or a combination of them [35–38]. These patients were treated with chemotherapy [29, 30, 34, 38], radiation therapy [31], chemo-radiation therapy [32], surgery [33, 39]. Some patients were treated differently within the trials [36, 37], while others had no treatment records [35]. QOL was evaluated using the EORTC QLQ-C30 [29–33, 36, 38], FACT-G [34, 35], or SF-36 [37, 39]. The follow-up period ranged from 8.3 months [31] to 5 years [32], although this was not described in two studies [34, 38]. Confounders in multivariate analysis were, in addition to the generally used variables, age, sex, body mass index, Eastern Cooperative Oncology Group Performance Status, smoking status, stage, comorbidities, and medical history, as well as the extent of resection [39], diffusing capacity of the lung for carbon monoxide [33] for surgery, PD-L1 expression level [30] for anticancer drugs, number of distant metastases, and history of brain metastases [34].

**Table 1:** Characteristics of the included studies

Author, Year	Country	Patients (number, sex, age)	Histology, Stage, Treatment	QOL	QOL domains	Follow-up period	Confounders	Number of deaths
Badaoui S et al., [30]2022	Australia	*n* = 2892 Female, 33% Age: 64 (58–70) years	NSCLC, Stage IV, Chemotherapy	EORTC QLQ-C30	Global Physical Emotional Role Cognitive Social	Median: 18 months	age, sex, race, ECOG-PS, smoking history, histology, PD-L1 expression level, comorbidity	Not reported
Erdem R et al., [29] 2022	Turkey	*n* = 50 Female, 8% Age: 55.9 (Min: 36, Max: 80) years	NSCLC, Stage IIIB and IV, Chemotherapy	EORTC QLQ-C30	Global Physical Emotional Role Cognitive Social	single-agent chemotherapy: 272 days combination chemotherapy: 445 days	Single or combination chemotherapy, Constipation, other QOL domains, Fatigue, Dyspnea, Insomnia	Not reported
Fielding R et al., [35] 2007	Hong Kong	*n* = 358 Female, 24.3% Age: 64.81 ± 10.28 years	SCLC *n* = 39 NSCLC *n* = 288 bronchogenic carcinoma *n *= 31, Stage 1- IV, Not reported	FACT-G	Global Physical Emotional Social	25 months	Histology, sociodemographic variables	*n* = 246
Langendijk H et al., [31] 2000	Netherlands	*n* = 198 Female, 15% Age: 0–70 years, 51%	NSCLC, Stage 1- IIIB, Radiation therapy	EORTC QLQ-C30	Global Physical Emotional Role Cognitive Social	Median survival 0 risk factors: 16.2 months 1 risk factor: 10.9 months 2 risk factors: 8.3 months	N-classification, weight loss and WHO performance status	Not reported
Movsas B et al., [32] 2009	USA	*n* = 239 Female, 31.8% Age: ≦70 years (70.7%) > 70 years (12.9%)	NSCLC, Stage II- IIIB, Chemoradiation therapy	EORTC QLQ-C30	Global Physical	5 years	age, treatment arm, karnofsky PS, histology, gender, tumor location, martial status, race, AJCC stage, hemoglobin, smoking status	Not reported
Möller A et al., [39] 2012	Sweden	*n* = 141 Female, 54% Age: 66.6 ± 9.1	NSCLC n,114 Carcinoid *n* = 15 Other *n* = 12, Stage 1- III, Surgery	SF-36	Physical Role Social	3 years	age, gender, comorbidities, extent of resection, tumor stage, smoking status, postoperative complications,	*n* = 53
Nieto-Guerrero Gómez JM et al., [36] 2020	Spain	*n* = 437 Female, 14% Age: 66 (range 31–88) years	NSCLC *n* = 297 SCLC *n* = 122 Others *n* = 18, Stage 1- IV Surgery *n* = 51, Radiation Therapy *n* = 378 Chemotherapy *n* = 382 Chemoradiation therapy *n* = 163	EORTC QLQ-C30	Global Physical Emotional Role Cognitive Social	30 months (range 7–76)	other QOL domains	Not reported
Pinheiro LC et al., [37] 2018	USA	*n* = 535 Female, 50% Age: 75 years	NSCLC *n* = 484 SCLC *n* = 51 Local *n* = 182, Regional *n* = 182, Distant/unknown *n* = 171, Surgery *n* = 241 Radiation therapy *n* = 182	SF-36	Physical Role Social	2 years	MCS, PCS, Physical function, General health, Role physical, Role emotional, Mental health, Social function, Body pain, Vitality	*n* = 300
Pompili C et al., [33] 2022	UK	*n* = 388 Female, 51% Age: 68.9 ± 9.6 years	NSCLC, pathologically R0 resections, Surgery	EORTC QLQ-C30	Global Physical Emotional Role Cognitive Social	Median follow-up: 55 months (IQR: 42–66)	age, BMI, gender, pathologic stage, DLCO	*n* = 120
Reck M et al., [34] 2012	Germany	*n* = 238 Female, not reported Age: not reported	SCLC, Extensive disease, Chemotherapy	FACT-G	Physical	Not reported	FACT-G and FACT subscale scores, age, gender, lactate dehydrogenase, number of metastatic sites, history of brain metastases, and ECOG PS	*n* = 87
Trejo MJ et al., [38] 2020	Australia	*n* = 111 Female, 45% Age: 64 (34–80) years	NSCLC *n* = 106 SCLC *n* = 5, Stage III and IV, Chemotherapy	EORTC QLQ-C30	Global Physical Emotional Role Cognitive Social	Not reported	sex, age, randomization arm	Not reported

*BMI* Body mass index, *DLCO* Diffusing capacity of the lung for carbon monoxide *ECOG* Eastern Cooperative Oncology Group, *EORTC QLQ-C30* European Organization for Research and Treatment of Cancer QLQ-C30, *SF-36* MOS Short-Form 36-Item Health, *FACT-G* Functional assessment of cancer therapy-general, *SCLC* Small cell lung cancer, *NSCLC* Non-small cell lung cancer, *IQR* Interquartile range, *MCS* Mental component summary, *PCS* Physical component summary, *WHO* World Health Organization

### Risk of bias assessment

The risk of bias was assessed using the Newcastle–Ottawa scale. Among the included studies, five were considered of high quality (8 or 9 points), and six were of moderate quality (6 or 7 points). Details are presented in Table 2.

**Table 2:** Quality assessment of included cohort studies using the Newcastle–Ottawa scale in systematic review and meta-analysis

Reference	Selection				Comparability	Outcome			Score
	Representativeness of the exposed cohort	Selection of the non-exposed cohort	Ascertainment of exposure	Demonstration that outcome of interest was not present at start of study	Comparability of cohorts on the basis of the design or analysis	Assessment of outcome	Was follow-up long enough for outcomes to occur	Adequacy of follow up of cohorts	Total
Badaoui, 2022 [30]	1	1	1	1	2	1	1	0	8
Erdem, 2022 [29]	1	1	1	1	0	1	1	0	6
Fielding, 2007 [35]	1	1	1	1	0	1	1	1	7
Langendijk, 2000 [31]	1	1	1	1	1	1	1	0	7
Movsas, 2009 [32]	1	1	1	1	2	1	1	0	8
Möller A, 2012 [39]	1	1	1	1	2	1	1	1	9
Nieto-Guerrero Gómez, 2020 [36]	1	1	1	1	1	1	0	0	6
Pinheiro, 2018 [37]	1	1	1	1	0	1	1	1	7
Pompili, 2022 [33]	1	1	1	1	2	1	1	1	9
Reck, 2012 [34]	1	1	1	1	0	1	1	0	6
Trejo, 2020 [38]	1	1	1	1	2	1	1	1	9

### Impact of global QOL on mortality risk

**Fig. 2:**
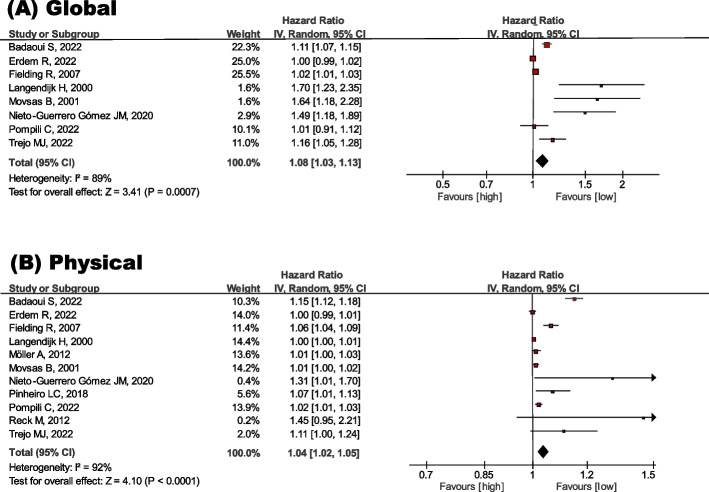
Meta-analysis for the effect of global and physical QOL on mortality risk

**Fig. 3:**
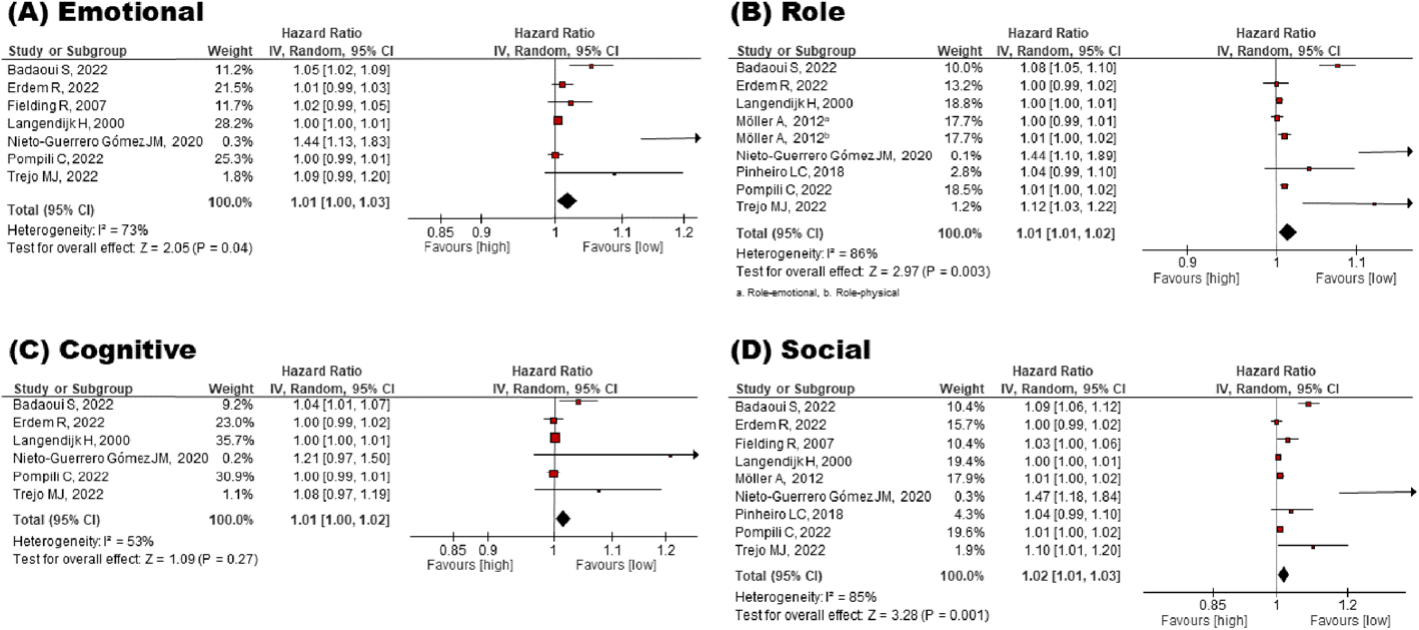
Meta-analysis for the effect of emotional, role, cognitive, and social QOL on mortality risk

The effect of QOL on the mortality risk was estimated using a forest plot of the inverse HR and 95% CI. Global, physical, social, role and emotional QOL were significantly associated with mortality risk, which was proven as follows: Global QOL (HR = 1.08 95% CI = 1.03–1.13) (Fig. 2), physical QOL (HR = 1.04 95% CI = 1.02–1.05) (Fig. 2), social QOL (HR = 1.02 95% CI = 1.01–1.03) (Fig. 3), role QOL (HR = 1.01 95% CI = 1.01–1.02) (Fig. 3), emotional QOL (HR = 1.01 95% CI = 1.00–1.0) (Fig. 3). In contrast, cognitive QOL was not significant: HR = 1.01 95% CI = 1.00–1.02 (Fig. 3).

## Discussion

This meta-analysis focused on the association between pretreatment health-related QOL and mortality risk in patients with lung cancer. The main findings of this study are summarized as follows: global QOL, physical QOL, emotional QOL, role QOL, and social QOL before treatment were factors affecting patient prognosis, and only cognitive QOL was not a significant factor.

Previous studies on the relationship between QOL and survival have debated whether global or physical QOL is a better predictor of survival. Zikos et al. [20] and Quinten et al. [17] reported that physical QOL is a superior predictor of survival. By contrast, Ediebah et al. [40] reported that global QOL was the strongest prognostic factor. In our study, HR for global QOL was proven to be the highest, followed by physical QOL.

However, it cannot be conclusively asserted that global QOL significantly predicts survival compared with physical QOL. Furthermore, when aiming to capture QOL not solely in terms of predicting survival but also to unveil the patient's vulnerability, the interpretation of global QOL results can be challenging due to its comprehensive nature. Considering this, we are inclined to believe that physical QOL holds more significance in illustrating the deterioration of a patient's physical function and establishing access to early intervention.

Regarding the relationship between physical functioning and QOL in cancer patients, a positive correlation has been reported between physical activity levels [41–43] and respiratory function [43]. However, there was a negative correlation between the performance status [44] and sedentary time [45]. Moreover, exercise tolerance [46, 47], physical activity levels [48], grip strength [49], and sarcopenia [50–52] are independent prognostic factors, indicating that physical QOL before treatment often reflects a decline in physical function. This strongly supports the importance of early exercise interventions soon after diagnosis. Previous reports have indicated that exercise counseling and intervention for lung cancer patients from the early stages of diagnosis improves their physical function and QOL [53–55]. It is necessary to establish exercise prescriptions based on patients' physical functions and verify whether these exercise prescriptions prolong not only physical function, but also survival time.

However, the focus of previous studies has only been on global and physical QOL. Indeed, in the analyses by Ediebah et al. [40] and Quinten et al. [17], functional QOL was a significant predictor of overall survival in the univariate analysis, whereas only global QOL and physical QOL were used in the multivariate analysis. Although these multivariate analyses may be useful in that they avoid multicolinearity and predict the domains of QOL that are most strongly associated with prognosis, this may be the cause of the lack of consideration of social, emotional, role QOL and prognosis of lung cancer patients. By contrast, this study reported novel findings on a significant association between social, role, and emotional QOL and mortality risk. Social, emotional, and role QOL are interrelated with psychological factors, including anxiety and depression [56–60], support from family and friends [61, 62], work and financial problems [63, 64], and symptoms, including dyspnea, fatigue, and appetite loss [8, 65, 66], which would lead to outcomes relevant to prognosis, such as access to medical care and physical function deterioration. Although the HR may be small, the results of this study suggest the need to consider the possible prognostic relevance of these factors.

In the present study, cognitive QOL alone was not significantly associated with survival. Numerous studies on cognitive QOL in patients with lung cancer have reported less decline compared to other functional QOL [67–69]. This is likely because there is little variation in cognitive QOL values and that patients with cognitive decline tend to be excluded from clinical trials [60, 68].

A comprehensive and individualized view of QOL is needed when confronting patients with lung cancer because social, emotional, and role QOL are related to prognosis in addition to global and physical QOL. First, a comprehensive assessment of QOL and domain-specific scores should be obtained. If there are physical QOL issues, this should be addressed based on the exercise prescription described above. In addition, comprehensive and individualized interventions are needed when there are social, emotional, and role QOL issues. Specifically, early social work, taking into account family, employment and social background, and psychological support from specialists are necessary. Such interventions may lead to functional and symptomatic improvements associated with QOL, which in turn may have a positive impact on treatment outcomes and prognosis. To explore better interventions to improve QOL and prolong survival, future research should focus on the social, emotional, and role QOL of patients with lung cancer before treatment to identify factors that affect QOL and further examine the effects of interventions on these factors.

This study had several limitations. This study integrated three different QOL assessments including EORTC QLQ-C30, SF-36, and FACT-G. Based on the previous study showing the correlation between the EORTC QLQ-C30 and the SF-36, which measure similar dimensions of QOL in patients with cancer [70], several QOL measures were integrated and analyzed. However, previous studies have demonstrated that QOL measures have reported low to moderate correlations between domains [71, 72]. Therefore, it cannot be excluded that differences in QOL measures may have influenced the results of this study, and this is the first limitation of this study. Second, the present study did not discuss symptoms, as in previous studies, because the symptom QOL was not included in the analysis. Third, it was not possible to perform a subset analysis based on the disease stage and treatment because of the number of selected articles. Finally, there was a gap of 20 years or more between the studies adopted. Particularly for medicinal treatments with anticancer drugs, the difference in survival time before and after molecular targeted therapy may potentially influence the results.

## Conclusion

We found that social, emotional and role QOL before treatment, not limited to Global QOL or physical QOL before treatment, were associated with mortality risk in patients with lung cancer. These results demonstrate the importance of comprehensive assessment of QOL and domain-specific scores to support patients with lung cancer. In addition to exercise prescription for physical QOL, early social work, taking into account family, employment and social background, and psychological support from specialists are necessary to improve QOL and prognosis.

### Supplementary Information

**Supplementary Material 1.**

## Data Availability

All data generated or analysed during this study are included in this published article and Supplementary appendix.

## Abbreviations

QOL: Quality of life
EORTC QLQ-C30: European Organization for Research and Treatment of Cancer Quality of Life Questionnaire-Core 30
SF-36: Medical outcomes study 36-item short-form health survey
CINAHL: Cumulative Index to Nursing and Allied Health Literature
FACT-G: Functional Assessment of Cancer Therapy-General
HR: Hazard ratio
CI: Confidence interval
WHO: World Health Organization
