# Correction: Pretreatment quality of life and survival in patients with lung cancer: a systematic review and meta-analysis

**DOI:** 10.1186/s12885-024-12299-2

**Published:** 2024-04-25

**Authors:** Taro Okayama, Katsuyoshi Suzuki, Shinichiro Morishita, Junichiro Inoue, Takashi Tanaka, Jiro Nakano, Takuya Fukushima

**Affiliations:** 1 Division of Rehabilitation Medicine, Shizuoka Cancer Center, Shizuoka, Japan; 2 Department of Physical Therapy, School of Health Science, Fukushima Medical University, Fukushima, Japan; 3 Division of Rehabilitation Medicine, Kobe University Hospital International Clinical Cancer Research Center, Kobe, Japan; 4 Department of Rehabilitation, Hyogo Medical University Hospital, Nishinomiya, Japan; 5 Faculty of Rehabilitation, Kansai Medical University, Osaka, Japan

**Correction** **:** **BMC Cancer 24, 495 (2024)**

**https://doi.org/10.1186/s12885-024-12267-w**

Following publication of the original article [1], the author reported a typesetting error, whereby Fig. 2 was erroneously duplicated as Fig. 3 in the published version. The publishers apologise for this error. The correct Fig. 2 and Fig. 3 are supplied in this correction article. The original article [1] has been corrected.

**Fig. 2:**
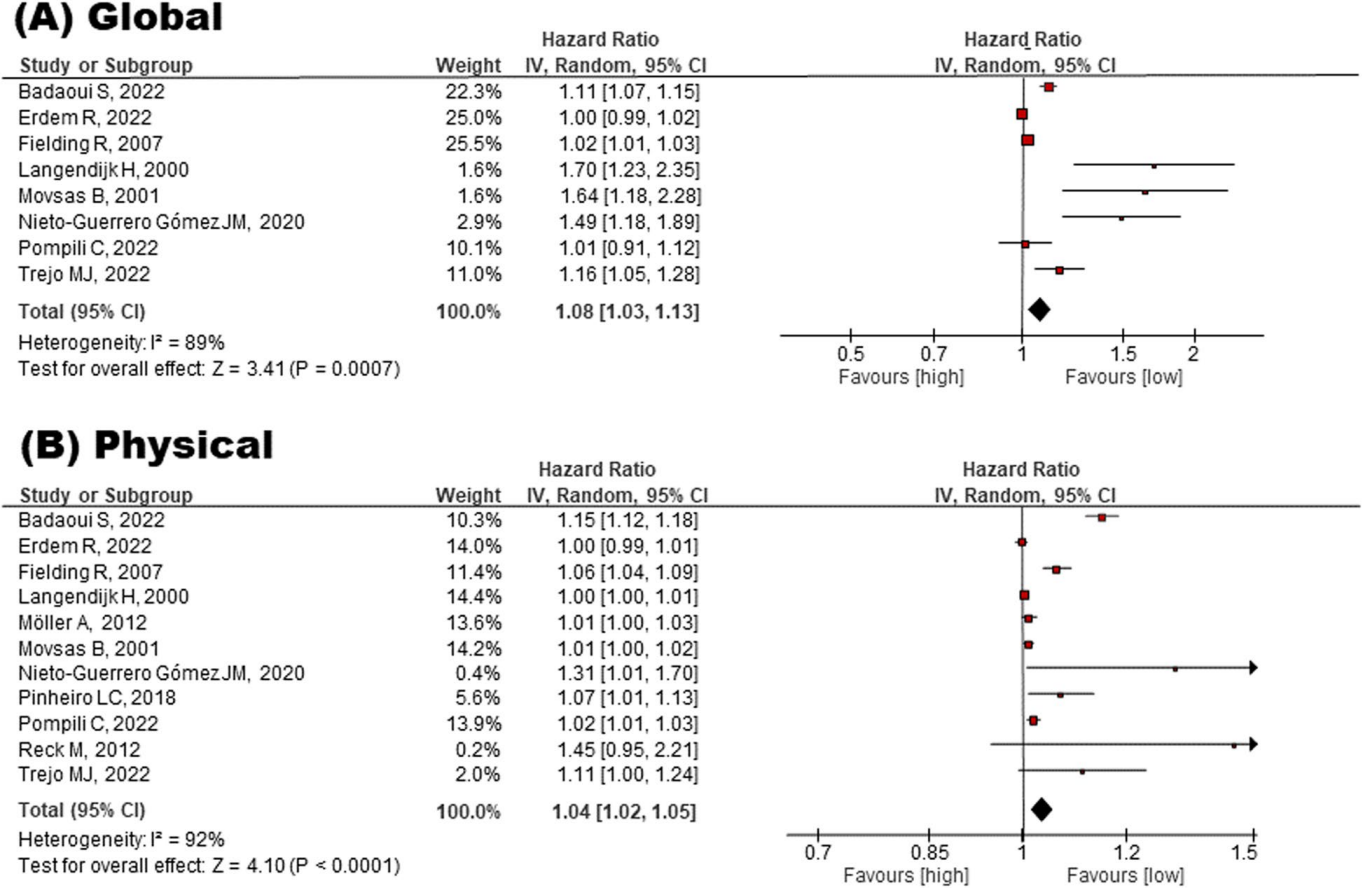
Meta-analysis for the effect of global and physical QOL on mortality risk

**Fig. 3:**
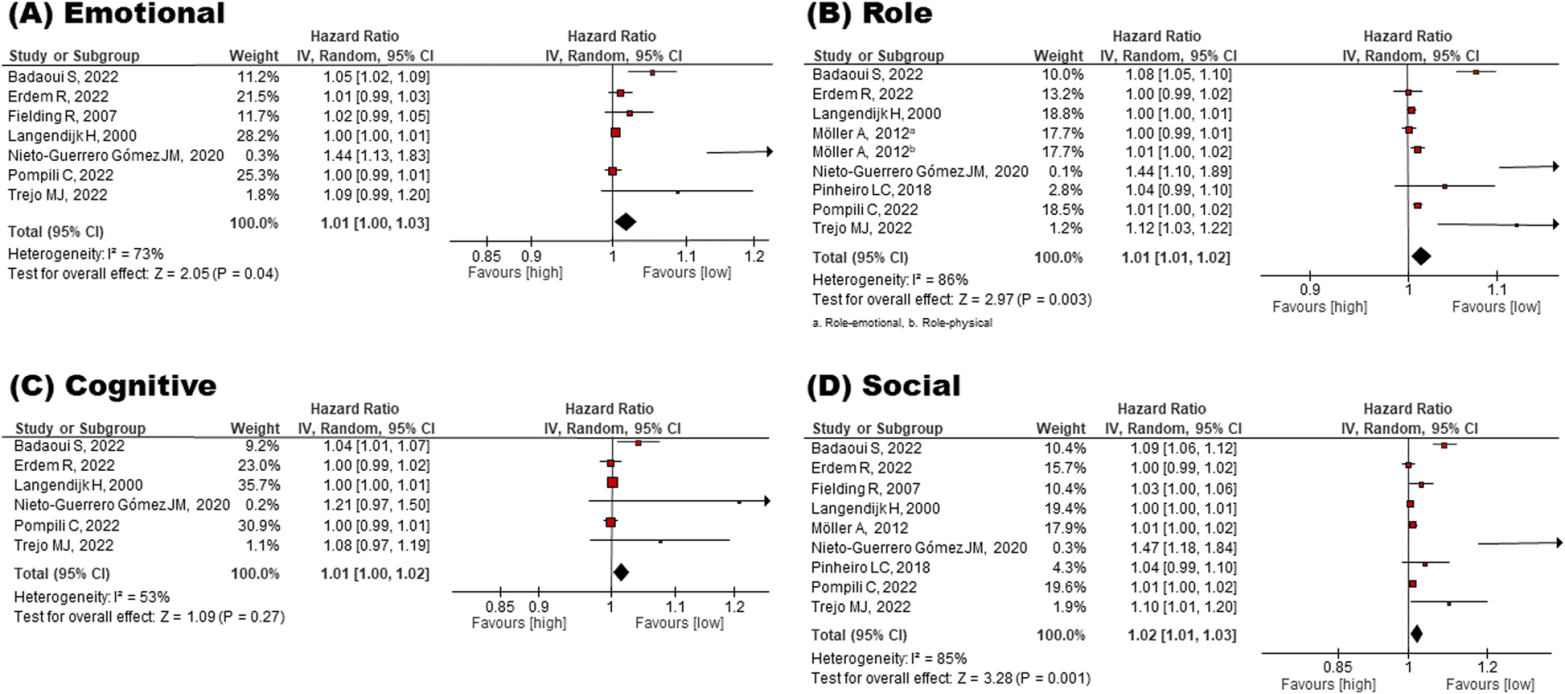
Meta-analysis for the effect of emotional, role, cognitive, and social QOL on mortality risk
