# Supplementary material for: Pretreatment quality of life and survival in patients with lung cancer: a systematic review and meta-analysis
Source: BMC Cancer. 2024 Apr 18;24:495. doi: 10.1186/s12885-024-12267-w (PMC11027547; doi:10.1186/s12885-024-12267-w)
Supplement: Supplementary file 1 — Supplementary Material 1. [file 12885_2024_12267_MOESM1_ESM.docx]

**Supplementary Appendix**

**Search strategy**

**PubMed/MEDLINE**

*((cancer OR neoplasm OR tumor OR sarcoma OR　hematological malignancy" OR lymphoma OR carcinosarcoma　OR leukemia) AND ("Quality of Life" OR QOL OR "EORTC QLQ　C30" OR SF-36 OR FACT-G OR "Functional Assessment of Cancer Therapy-General")) AND (mortality OR survival OR relapse OR recurrence)*

**CINAHL**

*(cancer OR neoplasm OR tumor OR sarcoma OR　hematological malignancy" OR lymphoma OR carcinosarcoma OR leukemia) AND ("Quality of Life" OR QOL OR "EORTC QLQ　C30" OR SF-36 OR FACT-G OR "Functional Assessment of Cancer Therapy-General") AND (mortality OR survival OR relapse OR recurrence)*

**Scopus**

*(cancer OR neoplasm OR tumor OR sarcoma OR “hematological malignancy” OR lymphoma OR carcinosarcoma OR leukemia) AND (“Quality of Life” OR QOL OR “EORTC QLQ C30” OR SF-36 OR FACT-G OR “Functional Assessment of Cancer Therapy-General”) AND (mortality OR survival OR relapse OR recurrence)*
